# Experimental and numerical investigation to optimise liquid desiccant system for advanced air conditioning

**DOI:** 10.1038/s41598-025-88738-2

**Published:** 2025-02-28

**Authors:** K. V. Shivaprasad, Sumit Roy, Alessandro Giampieri, Andrew Smallbone, Anthony Paul Roskilly

**Affiliations:** 1 Department of Engineering, Durham University, DH1 3LE Durham, UK; 2 Centre for E-Mobility and Clean Growth, Coventry University, CV1 2TL Coventry, UK

**Keywords:** Liquid desiccant dehumidification, Thermochemical network, Artificial neural network, Air conditioning., Environmental sciences, Energy science and technology, Engineering

## Abstract

This study aims to experimentally demonstrate a liquid desiccant systems effectiveness by using thermo-chemical fluid, such as aqueous solution of calcium chloride. This study evaluated the effect of operating temperatures on air properties (temperature, relative humidity, and moisture content) and system effectiveness by varying air flow rates. The system’s functionality was influenced by the operational temperature and air flow rate, and the dehumidification effectiveness was higher at low operating temperatures and low airflow rates. An ANN metamodel-based control strategy is also proposed for implementation in hybrid thermo-chemical networks with the help of system performance data and real-time data. The suggested ANN model’s results were validated using a variety of measuring techniques, including the RMSE, MAPE, correlation (R), and coefficient of determination (R^2^). The proposed ANN analysis achieved an excellent correlation between predicted and experimentally measured data.

## Introduction

In recent years, air conditioning systems have become an essential component of human life^1^. Air conditioning systems play a critical role in maintaining indoor air quality and ensuring occupant comfort. High humidity in the indoor environment can cause health problems, and hence, humidity control is a critical aspect of air conditioning systems. Air conditioning systems are responsible for a significant part of the world’s energy consumption, and their performance can have a substantial impact on the environment^2^. The excessive energy consumption of conventional systems for air conditioning has led to the development of alternative systems that are more energy-efficient and environmentally friendly^3,4^.

In recent decades, the expenditure of electrical energy has increased for the ventilation, air-conditioning, and heating sector due to the higher standards of human livelihood. This escalation of the energy consumed for heating, ventilation, and air conditioning (HVAC) caused a remarkable increase in demand for primary energy resources and destruct the electricity grid at peak times^2,5^. The design of buildings has a substantial influence on energy consumption, highlighting the need for more efficient HVAC solutions. Additionally, careful evaluation of moisture levels is essential during the design process of HVAC systems^6,7^.

Liquid desiccant systems (LDS) are among the most efficient and environmentally friendly alternatives that use a liquid desiccant to remove moisture in the air before it is cooled, reducing the energy required for cooling and improving indoor air quality^8^. In the LDS, the liquid desiccant absorbs the moisture in the air, and then the moisture-laden desiccant is regenerated to remove the moisture^9^. The regenerated desiccant is then reused to absorb moisture from the air. The use of liquid desiccant systems has several advantages, including lower energy consumption, reduced greenhouse gas emissions, and improved indoor air quality^9,10^. The effectiveness of the LDS using calcium chloride (CaCl_2_) as the desiccant has been extensively studied in recent years. However, the system’s performance is influenced by several factors, including air flow rate, operating temperature, and desiccant concentration^11,12^.

LDS has the benefits of being operated by the heat from renewable energy sources such as solar energy and the waste heat from industrial processes or power plants. The LDS has the capacity to accumulate energy and can be comfortably combined with a cooling unit to configure a hybrid system^13–16^. Giampieri et al.^17^ presented a review on the thermodynamics and economics of liquid desiccant systems. Similar in detail review studies on LDS systems have also been carried out by many researchers^18–21^. The physical characteristics of several liquid desiccant materials and their effect on the performance of the LDS system have been reviewed by Chen et al.^22^. The study confirms that CaCl_2_ is among the most often utilised liquid desiccants in the LDS due to its high affinity for water and low cost. Sahlot and Riffat^23^ briefly discussed the apparatus used in the LDS and the liquid desiccant’s characteristics. The performance control methods for various combinations of LDS and their influence on indoor air quality are summarised by Gurubalan et al.^3^. They also analysed the energy-saving potential of LDS with evaporative cooling depending on the climatic situations. Ronghui et al.^24^ studied the practical possibility of recent operations, including the component and material modifications for improving the liquid desiccant system performance. The various operational approaches and arrangements of LDS have been presented by Rafique et al.^25^. They briefly discussed the current status of desiccant-based evaporative cooling and also introduced some modified liquid desiccant evaporative coolers.

Numerous investigations have been conducted to evaluate the effectiveness of the LDS by using different liquid desiccants, such as CaCl_2_, lithium chloride (LiCl) and lithium bromide (LiBr)^26^. Among these, CaCl_2_ is the most economical and easily accessible desiccant for dehumidification. Abdul-Wahab et al.^27^ evaluated the dehumidifier performance in terms of effectiveness and moisture removal rate of the dehumidifier. The outcome of this investigation concluded that the air inlet temperature, flow rate, and concentration of desiccant are the most important variables in assessing dehumidifier effectiveness while air inlet temperature, desiccant inlet concentration, and flow rate are the predominant factors in forecasting moisture removal rate. An analogous experimental methodology was used by Jain et al. to examine the effectiveness of LDS. They also stated that the lower liquid-to-gas flow ratios result in higher deviations in effectiveness values^28,29^. Wang et al. designed an air-conditioning system utilizing a lithium chloride (LiCl) desiccant and evaluated its performance in reducing ambient air temperature and controlling humidity. Their findings demonstrated a temperature reduction of 17 °C, with specific humidity levels maintained between 12 and 13 g/kg^30^. Similarly, Srinivasan et al.^31^ conducted experiments to assess the performance of an LDS by varying the flow rate and concentration of calcium chloride (CaCl₂) desiccant solution. The study revealed that dehumidifier effectiveness improved with an increase in desiccant flow rate but decreased with higher air flow rates. Additionally, the results showed that moisture condensation rates increased with greater desiccant concentration and flow rate. Fekadu et al.^30^ reported that the use of calcium chloride (CaCl₂) as a desiccant in crossflow plate heat exchangers enhances the effectiveness of LDS. Further investigations analysed the performance parameters of LDS during regeneration and dehumidification processes by varying air velocity with two different concentrations of CaCl₂ desiccant. The results confirmed that variations in air flow rate significantly impact the overall effectiveness of the LDS system^33^.

The implementation of control strategies in LDS is essential to maintain optimal system performance, reduce energy consumption, and ensure occupant comfort. Hybrid thermo-chemical networks are developing technology that links different kinds of energy systems to optimise overall system performance. Recently, models of artificial neural networks (ANNs) have been utilised to forecast performance of photovoltaic (PV) and photovoltaic-thermal (PV/T) systems^34,35^. Al-Waeli et al.^36^ investigated the PV/T system which combines PV with a thermal collector and cooling systems with water, nano phase-change material (PCM), and nanofluid analytically and experimentally. An ANN based multi-layer perceptron (MLP) system has been used to analyse the system. The experiment has been carried out in an effort to confirm the results of the suggested ANN models. The suggested ANN technique demonstrated that the electrical efficiency of employing nanofluid/nano-PCM was raised from 8.07 to 13.32%, resulting in a 72% thermal efficiency.

The purpose of this study is to experimentally evaluate the effectiveness of a liquid desiccant system using CaCl_2_ as the desiccant by varying operating temperatures and air flow rates. Additionally, an ANN metamodel-based control strategy is proposed for implementing a hybrid thermo-chemical network with the help of real-time data. The ANN metamodel-based control strategy proposed in this study aims to utilise the ANN model’s predictions to optimise system performance and reduce energy consumption. The proposed strategy utilises real-time data to predict system performance and make control decisions, enabling the system to respond to changing indoor and outdoor conditions. This study is novel in integrating experimental findings with ANN based control strategy. Unlike previous works, this approach not only evaluates system performance under varying conditions but also proposes real-time optimization methods for hybrid thermo-chemical networks, bridging a significant research gap.

## Experimental setup

**Fig. 1:**
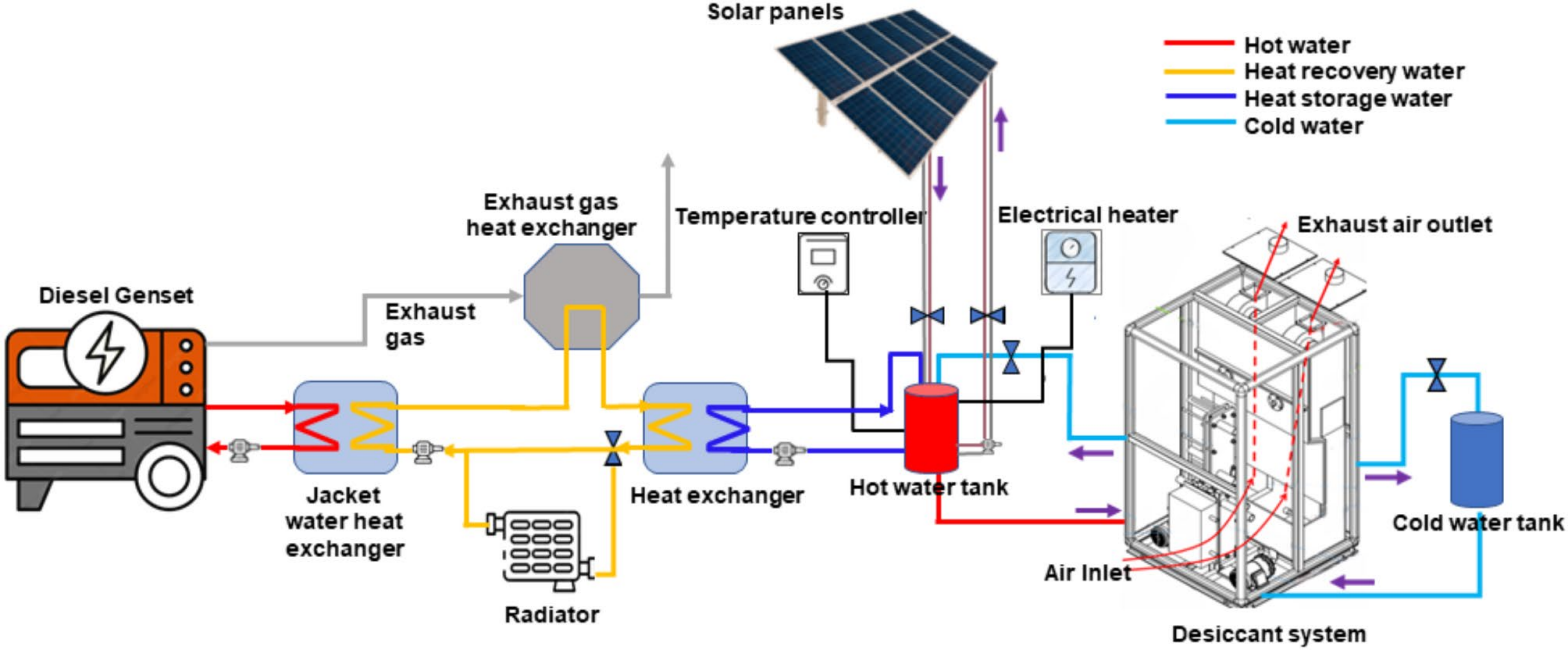
Schematic diagram of testing setup.

**Fig. 2:**
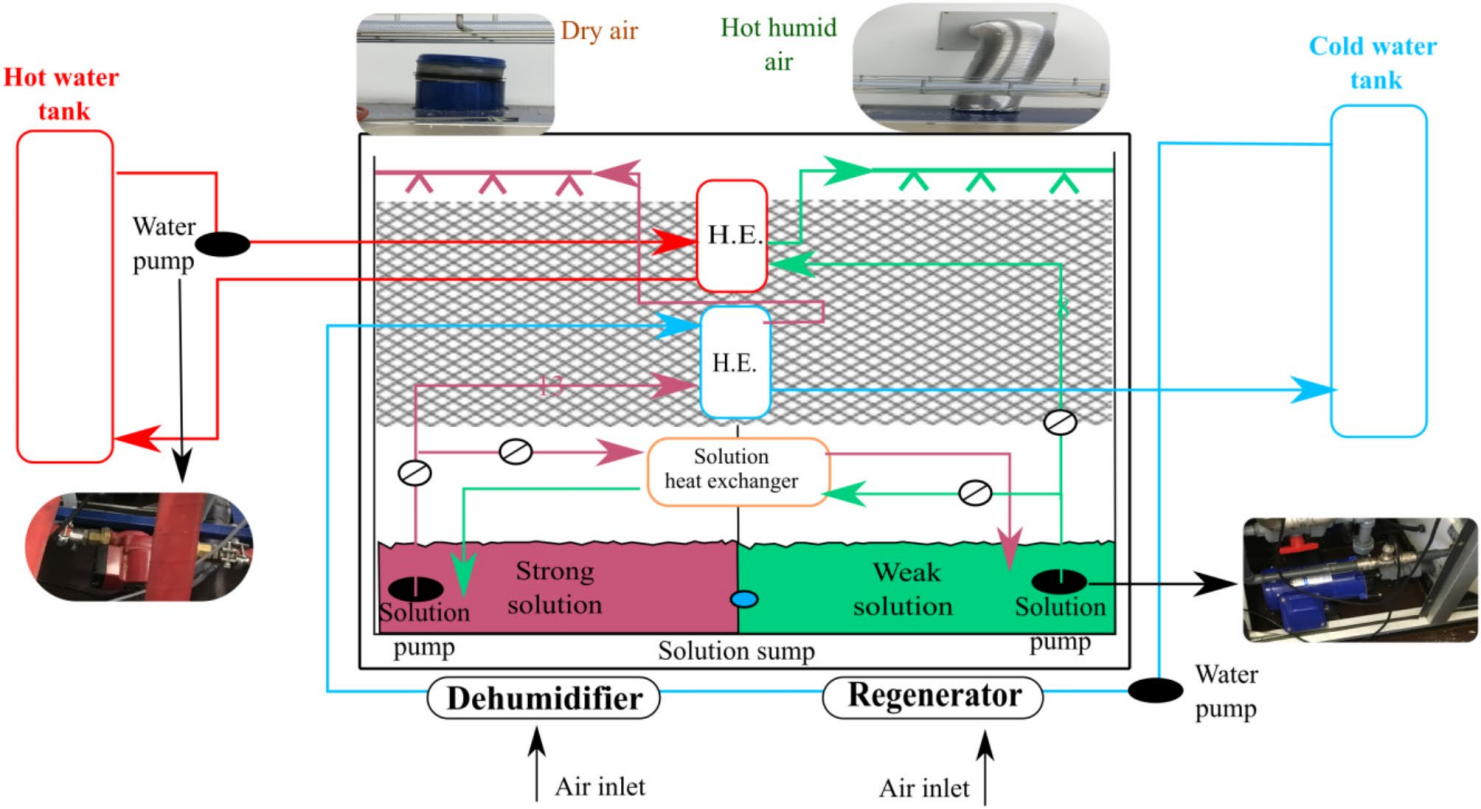
TCF flow in the system.

The schematic diagram of the thermo-chemical fluid (TCF) experimental test platform is presented in Fig. 1 and the flow of the TCF in the system is shown in Fig. 2. A combined heat and power (CHP) system is a hybrid configuration designed to optimise energy efficiency by integrating multiple components. The system consists of a Kohler SDMO 17.6 kW/22kVA three-phase diesel generator and a RS company-made fan heater. The fan heater is employed to control the desired engine load to reach the required water temperature. A diesel generator serves as the primary energy source, recovering waste heat via a jacket water heat exchanger and an exhaust gas heat exchanger. This recovered heat is utilised for preheating water or regenerating the desiccant solution. A radiator dissipates excess heat to ensure safe operation. Solar panels supplement the system with renewable energy, powering the temperature controller and electrical heater to reduce reliance on the generator. The hot water tank stores thermally elevated water, incorporating heat from the exchangers, solar panels, and electrical heater, with the temperature controller maintaining optimal performance.

The thermal-driven liquid desiccant dehumidifier developed by Nanjing Hanwei Nanleng Refrigeration Group Co. Ltd. The thermal-driven liquid desiccant dehumidifier has two heat exchangers, a chiller, humidifier, dehumidifier, cold water tank, hot water tank, and a container tank for liquid desiccant. A counter-flow energy recovery heat exchanger named recuperator is situated within the supply. An air blower is utilised to blow the ambient air through the dehumidifier and regenerator. The hot water tank’s water is used for dilute solution regeneration. Grundfos UPS2 magnetic motorised circulator pumps are used to pump the liquid desiccant across the system. The electrical control system uses the PLC technology, and the PLC outputs the signals to each executive modules, and control and operation of each execution mechanism. The heating source to produce hot water is attained by the recovered waste heat of the diesel engine, while the cooling source to cool the water in a cold-water tank is a chiller. MTA manufactured TAEevo chiller uses refrigerant R134a to lower the cold tank’s water temperature. The system incorporates a chiller utilizing refrigerant R134a to regulate the temperature of the cold-water tank, ensuring a consistent and optimal cooling environment for the desiccant system. The cold-water tank acts as a thermal reservoir, enabling precise temperature control during the dehumidification process. Additionally, water loops are employed to continuously circulate the desiccant solution between the heat exchanger and the dehumidifier. This circulation facilitates heat and mass transfer, allowing the desiccant solution to efficiently absorb moisture from the air in the dehumidifier while releasing the absorbed heat in the heat exchanger. These integrated components work together to enhance the system’s overall dehumidification performance and energy efficiency.

From the solution container, the desiccant is cooled in a solution heat exchanger before it circulates in the dehumidifier. In the dehumidifier, cooled concentrated desiccant absorbs the water vapour from air and thus gets diluted. The processed desiccant flows into the solution container and is then circulated to another heat exchanger where the hot water exchanges its heat with the diluted weak desiccant. The diluted hot desiccant then goes into the regenerator, which directly interacts with the atmospheric air. During this process, the water molecules in the diluted hot desiccant solution desorb into the air and get concentrated. The liquid desiccant solution must be cooled/heated before entering the dehumidifier/regenerator for the absorption/desorption process to happen. The dry air from the dehumidifier can be further cooled by evaporative cooling.

Table 1 provides the experimental test matrix of this study. The operating temperatures (30 °C, 45 °C, and 60 °C) were selected based on prior studies, which showed optimal system performance and energy efficiency at these levels^17^. The density of the solution was first measured by using a pycnometer. Once measured, the density and the temperature of the desiccant solution were used to ascertain its mass fraction (using the equation developed by Conde for the density of aqueous CaCl_2_^37^).

**Table 1:** Experimental test matrix.

Source of heating	Diesel engine heat
Operating temperature of heating water	60 °C, 45 °C, 30 °C
Operating velocity of air	17 m/s, 12 m/s and 5 m/s
Liquid desiccant solution concentration	Aqueous CaCl_2_ (40.5% wt.)
Evaluation parameters	Change in temperature, relative humidity, and moisture content of air Dehumidification effectiveness

### Performance parameters

The liquid desiccant system’s performance is calculated on the basis of the dehumidification effectiveness and the moisture removal rate.

#### Dehumidification effectiveness

The ratio between the actual change in the moisture level in the air across the dehumidifier and the ideal change that a desiccant solution can achieve at a fixed temperature and mass fraction is known as the dehumidification effectiveness, *ε*_*deh*_. This parameter can be calculated using Eq. (1)^38^:

1 $$\:{\epsilon\:}_{\text{d}\text{e}\text{h}}=\frac{{\omega\:}_{\text{a},\text{i}\text{n}}-{\omega\:}_{\text{a},\text{o}\text{u}\text{t}}}{{\omega\:}_{\text{a},\text{i}\text{n}}-{\omega\:}_{\text{e}\text{q},\text{s}\text{o}\text{l}}}$$

where *ω*_*a, in*_ and *ω*_*a, out*_ represent the moisture level in the air (kg_H2O_/kg_dry air_) at the inlet and outlet of the dehumidifier, while *ω*_*eq, sol*_ is the equilibrium moisture level of the desiccant solution (kg_H2O_/kg_dry air_) at the entry of dehumidifier, which can be calculated using Eq. (2)^39^:2

where *P*_*sol*_ is the desiccant solution’s equilibrium vapour pressure (kPa),  is the mass fraction of the desiccant salt in the solution (kg_salt_/kg_sol_) and *T* is the desiccant solution’s temperature (°C).

#### Moisture removal rate

The quantity of moisture removed from the air stream by the desiccant solution per unit of time is known as the moisture removal rate, *MRR* (kg/s), and it can be determined by using Eq. (3)^40^:

3 $$\:{MRR=\:m}_{\text{f}\text{a}}\left({\omega\:}_{\text{a},\text{i}\text{n}}-{\omega\:}_{\text{a},\text{o}\text{u}\text{t}}\right)$$

where *m*_*fa*_ is the supplied air’s mass flow rate to the dehumidifier (kg/s).

### Experimental uncertainties

To ensure the reliability of the experimental results, measurement uncertainties were carefully addressed using calibrated instruments with well-defined error margins. The flow rate was measured with an accuracy of ± 1%, temperature readings were taken with a precision of ± 0.5 °C, relative humidity (RH) was recorded with an uncertainty of ± 2%, and pressure measurements were conducted with an error margin of ± 0.1 bar. These calibrated instruments were selected to minimise systematic errors and enhance the accuracy of the data. Furthermore, multiple trials were conducted for each experimental condition to validate the consistency and reproducibility of the results. This rigorous approach to managing experimental uncertainties enhances the credibility and reliability of the reported results.

## Results from the experimental work

This section describes the effect of operating temperatures on air properties and system effectiveness in conjunction with the CHP system. Table 2 provides the ambient air conditions at operating temperature of 45°C for various flow rates.

**Table 2:** Ambient or initial air conditions.

Flow rate(m/s)	Initial air temperature (°C)	Relative humidity (%)
17	14.3	50.1
12	19.9	43.6
5	20.6	43.8

### Effect of operating parameters on ambient air properties

**Fig. 3:**
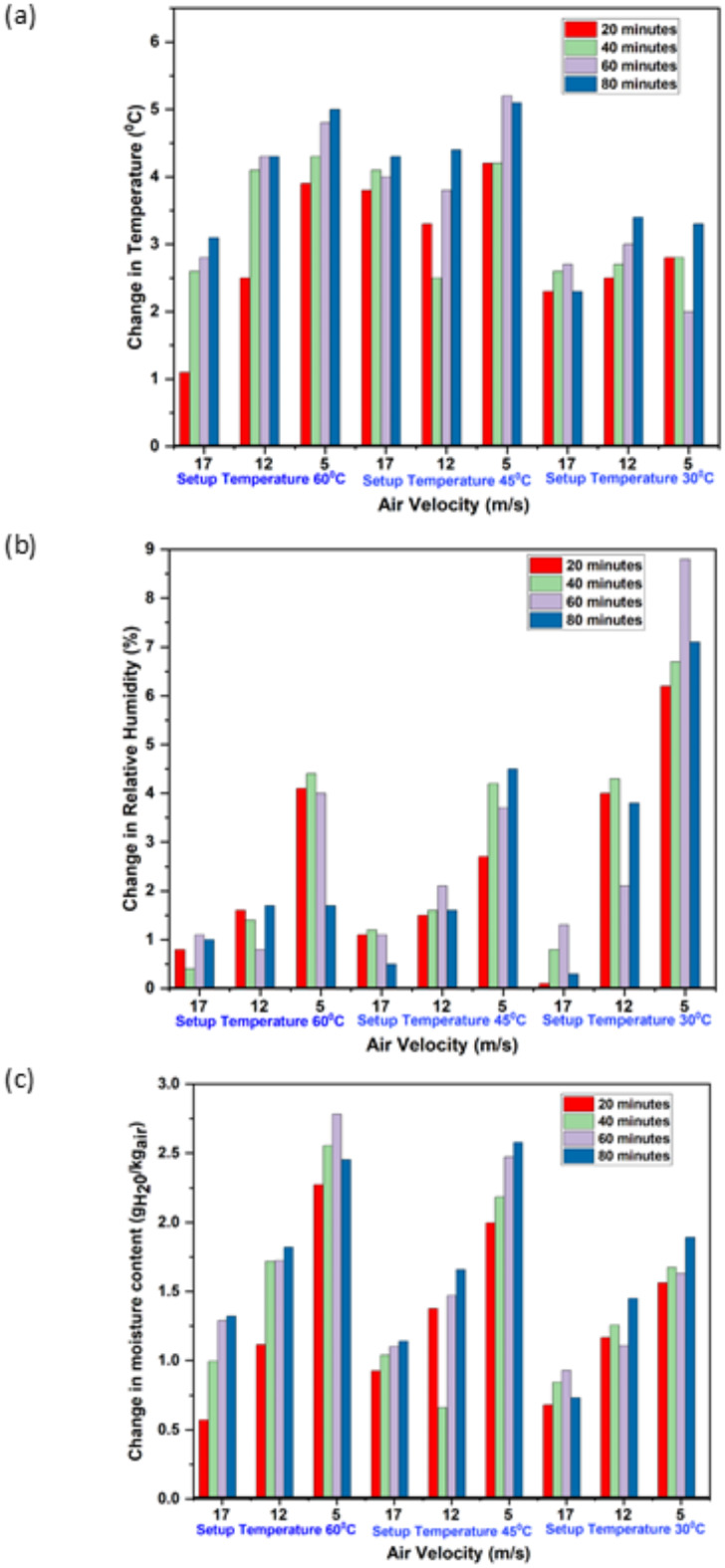
(**a**,** b** and** c**): Change in ambient air properties at various flow rates and temperatures.

Figure 3a and b illustrate the variation in temperature and relative humidity (RH) of ambient air at different flow rates (17 m/s, 12 m/s, and 5 m/s) under setup temperatures of 60 °C, 45 °C, and 30 °C over time. The results indicate that the maximum changes in temperature and RH occur at the lowest air flow rate of 5 m/s across all operating temperatures. This can be attributed to the increased contact time between the air and the solution at lower flow rates, which enhances heat and mass transfer efficiency. Conversely, as the air flow rate increases, the reduced contact time diminishes the effectiveness of heat and mass exchange, resulting in smaller changes in air properties.

The most significant change was observed at 45 °C for the 5 m/s flow rate, where the air temperature increased by a maximum of 5.2 °C and the moisture content varied within the range of 1.5–2.8g_H20_/kg_dry air_. This highlights the importance of optimal flow rates and operating temperatures in achieving efficient heat and moisture transfer. The findings suggest that lower flow rates, coupled with moderate temperatures like 45 °C, provide favourable conditions for maximising the changes in air properties, making this combination particularly effective for such processes.

The variation in relative humidity (RH) decreases as the air flow rate increases, regardless of the operating temperature. This is evident in Fig. 3b, which shows that the maximum change in RH of ambient air consistently occurs at the lower air flow rate of 5 m/s across all tested operating temperatures. This phenomenon can be attributed to the increased contact time at lower flow rates, allowing more effective moisture exchange between the air and the solution. In contrast, higher air flow rates reduce the interaction time, limiting the extent of moisture transfer and thereby diminishing RH variation. These results emphasise the critical role of air flow rate in optimising humidity control processes.

The moisture removal rate (MRR) depends on the mass flow rate and absorption capacity, exhibiting a positive relationship with the mass flow rate. However, as the air flow velocity increases, the MRR decreases due to diminished mass transfer efficiency caused by reduced contact time between the air and the solution. Figure 3c highlights the changes in moisture content of ambient air at various air flow rates (17 m/s, 12 m/s, and 5 m/s) across different setup temperatures (60 °C, 45 °C, and 30 °C). The plots clearly demonstrate that the greatest changes in moisture content occur at the lowest air flow rate of 5 m/s, regardless of the operating temperature. This trend underscores the critical role of maintaining lower flow rates to optimise mass transfer and achieve higher moisture removal from the air.

### Dehumidification effectiveness

**Fig. 4:**
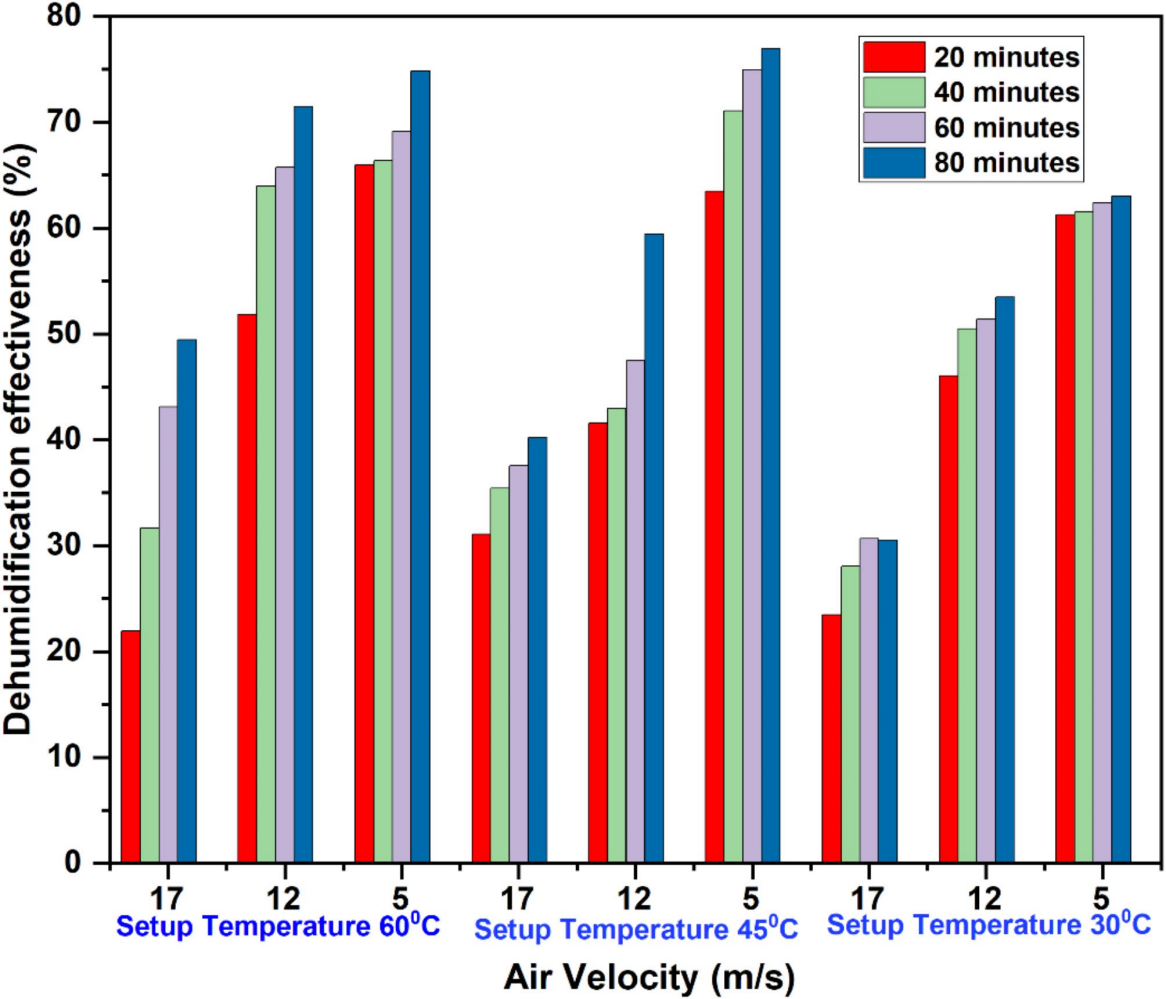
Experimental results of the dehumidification effectiveness.

Figure 4 shows the system’s dehumidification effectiveness at different mass flow rates and operating temperatures over a period. As seen from the plot, the system effectiveness is greater for lower air flow rates compared to higher flow rates irrespective of all operating temperatures. At a higher air flow rate, the humidity of the outlet air from the system is augmented owing to reduced contact time between the desiccant solution and air across the dehumidifier. Thus, the dehumidification effectiveness decreases when the air flow rate increases.

The system attained maximum effectiveness of 77% at 45 °C operating temperature and 5 m/s air mass flow rate compared to other operating temperatures. The system shows the least effectiveness in the range of 11–30.5% for the operating air flow rate of 17 m/s and 30 °C temperature. At 5 m/s air flow rate, the system produced dehumidification effectiveness of 74.8%, 77%, and 63% for the operating temperature of 60 °C, 45 °C, and 30 °C, respectively. The superior performance at 45 °C, compared to 60 °C, is due to enhanced desiccant regeneration efficiency and more favourable thermodynamic properties for moisture absorption. At 45 °C, desiccants regenerate effectively without excessive drying or degradation, maintaining their moisture-absorbing capacity. In contrast, at 60 °C, over-regeneration can reduce absorption efficiency and damage the desiccant. Additionally, the thermodynamic conditions at 45 °C ensure a balanced moisture absorption process, whereas higher temperatures like 60 °C can lead to reduced uptake capacity. From this, it can be concluded that the operating temperature of 45 °C and air flow rate of 5 m/s are effective operating parameters among other parameters to attain better dehumidification effectiveness.

##  Intelligent control algorithm for the thermo-chemical energy network

**Fig. 5:**
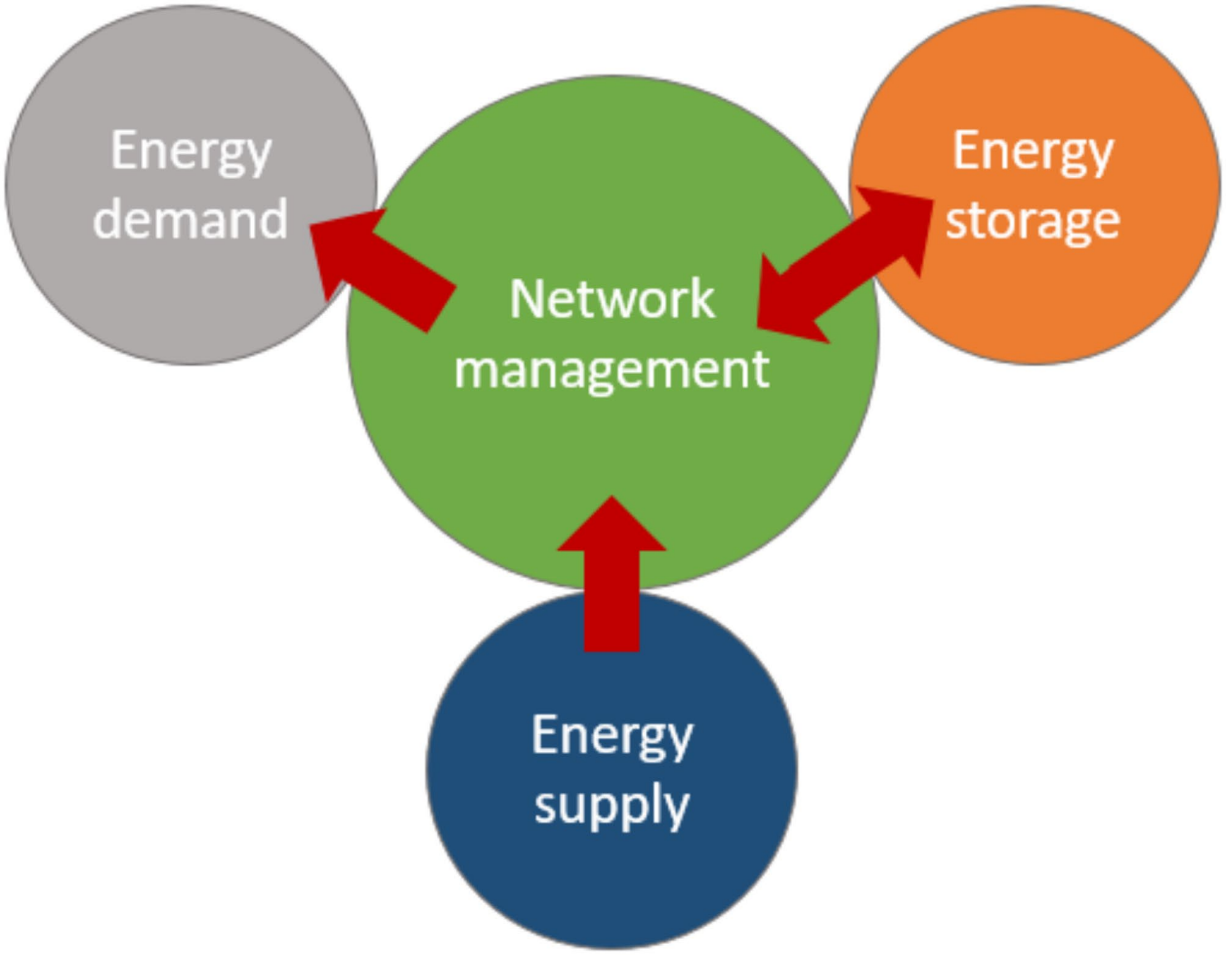
A schematic showing the main components of the energy system.

The proposed control algorithm for implementation in hybrid thermo-chemical networks is set out in this section. The active component of the system is set out in Fig. 5.

These can be summarised as:

1. **Energy supplier nodes** – sources of thermal energy used to recover the TCF. Typical examples of sources might be an excess heat source from an industrial process, excess heat from a combined heat and power system, a heater element or solar thermal energy. Sources could be intermittent in their supply.
2. **Energy demand nodes** – an energy demand requiring heating, cooling or drying services. Demands could be intermittent in their operational schedule but would largely be delivering or partially supporting local air-conditioning within buildings or industrial processes.
3. **Energy storage nodes** – two-way interaction with the network, managed storage of concentrated and weak TCF mixtures.
4. **A network system of edges** – a connected set of pipes and electrical measurement and actuation systems to support the management of the network. Energy demand and supply should be matched to ensure resilience and confidence by its users.

### Energy system balance

As with any energy system, net energy demand and supply across the network may not be balanced without a network management strategy. The cost of storage for TCF is low, thus any requirement for instantaneous balancing strategies is not required. Instead, extended periods of balancing have been considered.

The demonstrator has explored different network nodes and configurations. This process has led to the development of the proposed operational model for the real-time technical management of TCF network loading.

The TCF network carries a salt solution. Losses of salt from the solution are negligible over a daily operational cycle, thus the state of the system is based on the total water contained within the TCF across the network. This can be determined by measuring the volume contained in the two storage TCF tanks.

At each 30-minute interval, a centralised control system evaluates the following steps:

Step 1. Evaluate the current state of the system.
Step 2. For each node, estimate the demand schedule over the next 24 h.
Step 3. For each node, estimate the supply schedule over the next 24 h.
Step 4. If imbalanced, determine the timing to take appropriate action.

*Step 1: Evaluate the current state of the system*.

It is possible that the previous time period made an estimation of how much TCF would be used or be recovered and it was inaccurate. Furthermore, it is also possible that it could have been inaccurate for a several time steps.

The net-water balance is estimated according to currently observed water volumes in the two storage tanks. Additionally, the change in the expected and measured temperatures are determined in the last time step and its impact on recovery rates is estimated. The outcome is an estimate of the current state of the system, *E*_net_ in m^3^ of water.

If *E*_net_=0, it is ideal.

If *E*_net_>0, less demand or more supply is required to balance the system.

If *E*_net_<0, more demand or less supply is required to balance the system.

When not ideal, it will require an intervention by the control system in Step 4.

*Step 2: Estimate demand schedule*.

To ensure that the system has sufficient capacity to deliver the demand expected and if required to mitigate for this, an estimate of the system demand is required.

**Fig. 6:**
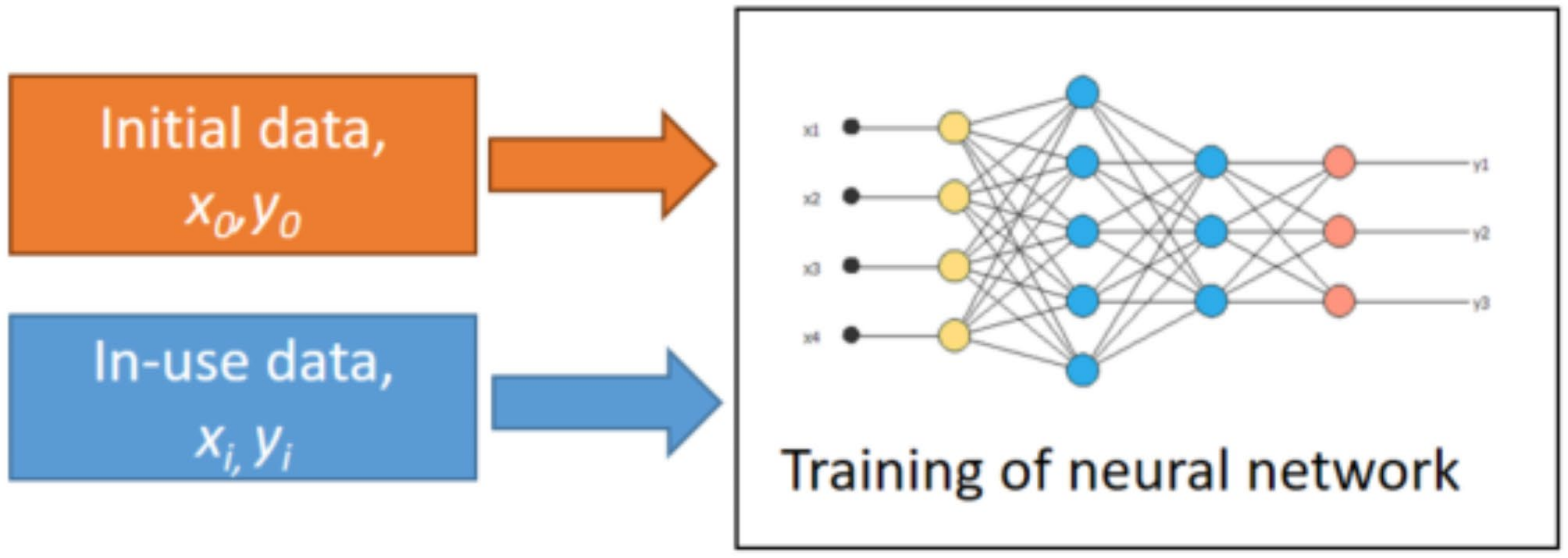
Neural network training.

Within the algorithm, the demand of each node is estimated over the next 24 h. As shown in Fig. 6, the basis of these estimates is framed around;

1. Historical data measurements, termed initial data or estimates of typical load depending on the daily operation of their energy demand. This could be from models, measurements, or other sources.
2. Real-time data from the system’s dynamic operation.

**Fig. 7:**
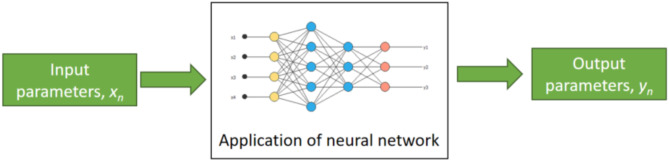
Application of neural network.

Key input parameters *x*, were used to construct and train a neural network and these input parameters can be applied (as shown in Fig. 7), to make data-driven estimates of node demand, *y* over the next time period.

This could be considered as a form of self-learning because it intelligently schedules the use of the system based on a neural network of system data. It also mirrors a smart-grid by offering demand and supply side responses to any supply/demand in-balance.

### Computational methodology

Polynomial Regression Models, Radial Basis Function, ANNs, Kriging, gene expression programming (GEP) and their respective variants represent the major classes of meta-modelling approaches among many others. Their robustness and success as alternative control systems in various disciplines as per their individual scope of appropriateness to a given problem at hand, have seen an unprecedented proliferation of their applications into the model-based control paradigms of the day.

In contrast to conventional metamodel-based control strategies, ANN models are capable of deriving nonlinear models directly from measurable input/output data, significantly lowering the expenses, development time, and complexity involved in developing control systems^41–43^.

Artificial intelligence-based modelling offers the potential for a very fast, multidimensional, adaptive model of the system under study. In addition to this, ANN model handles data with a considerable degree of observational uncertainty and its low computational costs that enable predictions and interpolations for control decisions^44^, . The intelligence systems based on artificial neural networks provide intrinsic adaptive strengths to model complex nonlinear systems at high computational speeds even when presented with noisy and incomplete data without any a priori knowledge of the physics of the issue under process, positioned themselves as a robust and sought mapping tool in the control domains. Early researchers were quick to recognise the potential of multi-layered ANN networks in incorporating nonlinear trends in complex data to fit arbitrarily complex nonlinear models to any required precision and demonstrate their inherent resilience as universal approximators^45,46^, .

**Fig. 8:**
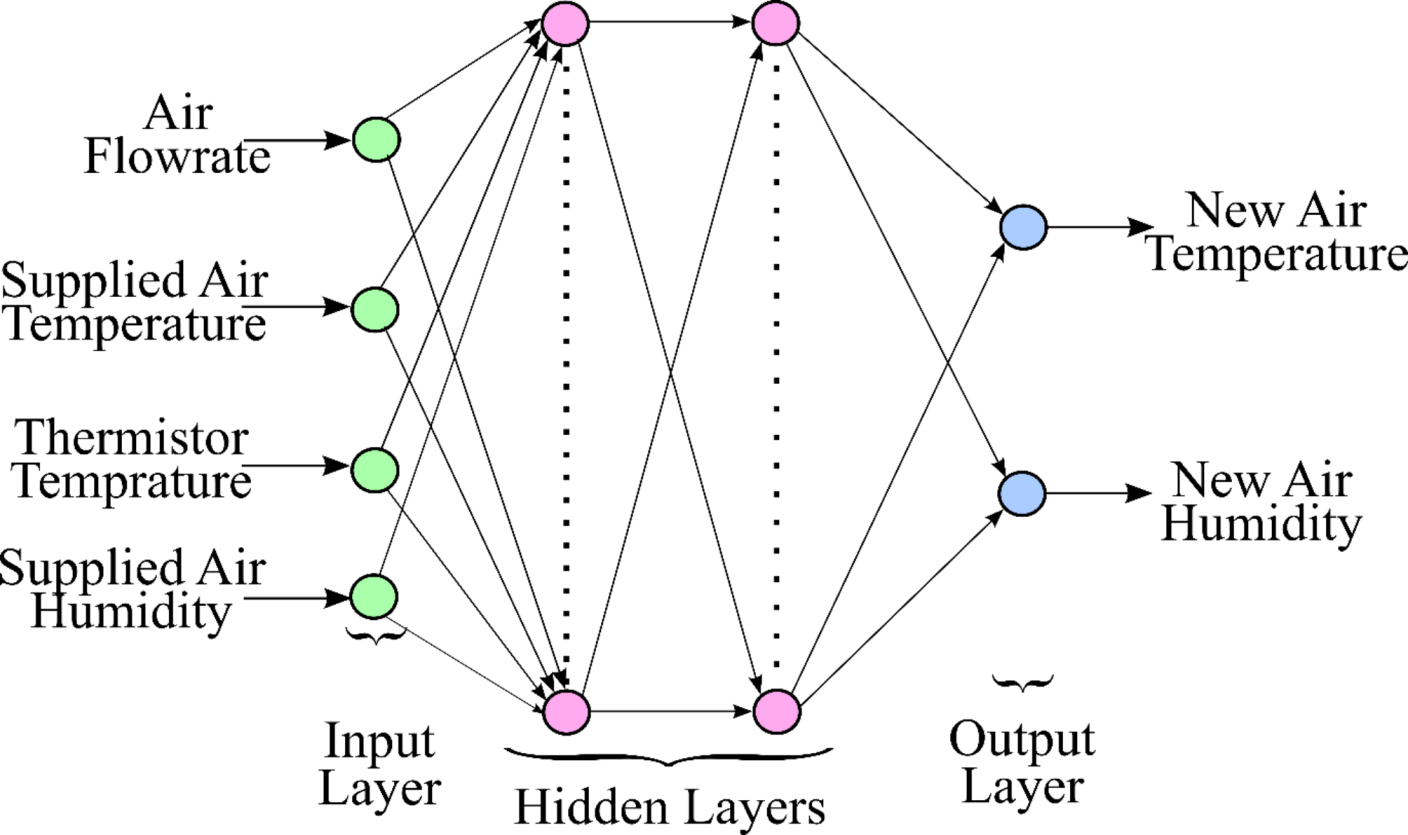
Architecture of the proposed ANN for CHP system.

Consequently, an ANN model was developed for the CHP system having air flowrate, supply air temperature, thermistor temperature, and air humidity as control variables and new air temperature and humidity as response variables (Fig. 8). A conventional multiple input multiple output (MIMO) architecture based on the Levenberg-Marquardt algorithm and log-sigmoid activation function was developed on the Matlab^®^v2022 software. Since mean square error (MSE) has the highly desirable characteristics of convexity, symmetry, and differentiability, MSE was selected as the loss function to be minimised. A topology of two (2) hidden layer with 8 hidden neurons in each layer for the given four (4) inputs and two (2) output was selected for the CHP system (Fig. 8). The ANN model was trained using 70% of the dataset, corresponding to 420 data points. The remaining 30% of the dataset was divided into 15% for validation (90 data points) and 15% for testing (90 data points). Table 3 illustrates the detailed network architectures of the developed models with its corresponding stopping criteria.

**Table 3:** Details of the ANN network parameters developed on the MATLAB platform.

Topology	4 inputs, 2 outputs, and 2 hidden layers with 8 hidden neurons in each layer (4-8-8-2)
Data	***Training subset***: 70% randomly selected observation data ***Validation subset***: 15% randomly selected observation data ***Test subset***: 15% randomly selected observation data
Activation function	Log-Sigmoid
Training algorithm	Levenberg-Marquardt
Loss function criteria	Minimum MSE
Stopping criteria	Stop the network training when the validation error starts increasing

To assess the prediction performance of the suggested ANN models, we have considered correlation coefficient (R) and coefficient of determination (R^2^). The R^2^ has been taken into consideration in order to assess the effectiveness of the ANN model, and it is defined by Eq. (4)^41^. The values of R^2^ above 95% and higher are considered that the model is significant.

4 $$\:{R}^{2}=1-\left(\frac{\sum\:_{i=1}^{n}\left({t}_{i}-{o}_{i}\right)}{\sum\:_{i=1}^{n}{\left({o}_{i}\right)}^{2}}\right)$$

where “t” represents the actual output, “o” stands for the predicted output value, and “n” represents the number of patterns in the data set.

Furthermore, we assessed our model using two statistical error metrics, namely mean absolute percentage error (MAPE) and root mean square error (RMSE). In the context of the literature, for the developed ANN model’s performance a limit of 5% for the MAPE has been taken for the output parameters. Equations (5) and (6) defines the RMSE and MAPE^47,48^:

5 $$\:RMSE=\sqrt{\frac{1}{n}\sum\:_{i=1}^{n}{\left({t}_{i}-{o}_{i}\right)}^{2}}$$

6 $$\:MAPE=\frac{1}{2}\sum\:_{i=1}^{n}\left(\left|\frac{{t}_{i}-{o}_{i}}{{o}_{i}}\right|\right)\times\:100$$

**Fig. 9:**
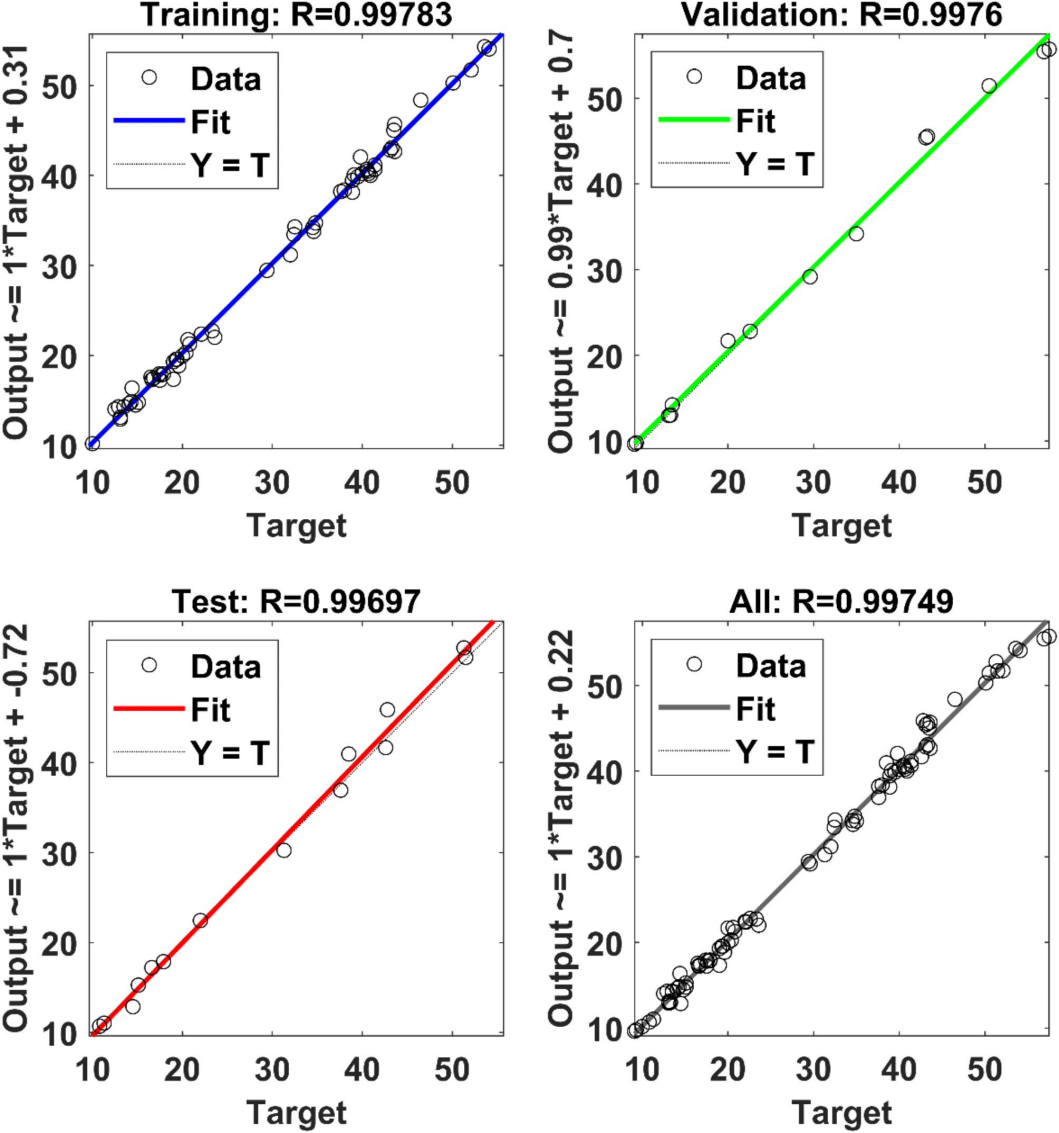
Overall correlation coefficients of the developed network for CHP system.

The comprehensive correlation coefficient ‘R’ of the selected network architecture is shown in Fig. 9. Inspection of the figure indicates the network’s projected values and actual observations exhibit impressive consistency throughout its operational range. This suggests the network’s inherent sensitivity and resilience in its capacity to map the output parameters with excellent accuracy.

**Fig. 10:**
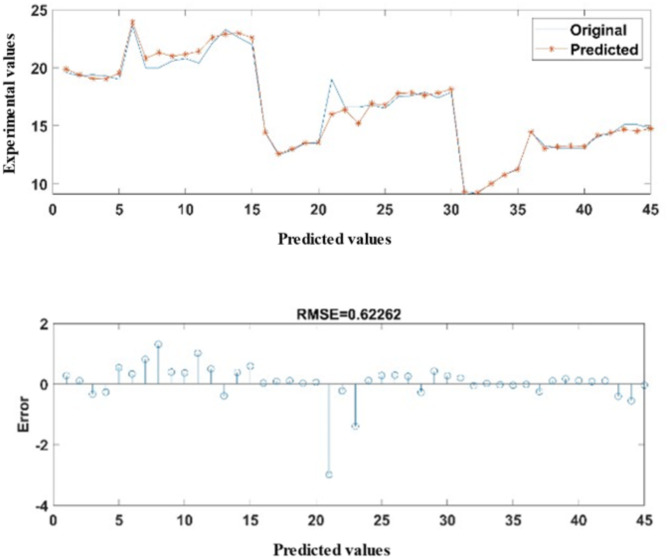
Comparison of experimentally measured data and ANN-predicted New Air Temperature.

Figure 10 illustrates the ANN predicted versus experimental data for New Air Temperature (NAT). According to the CHP system’s statistical analysis, the developed ANN model for NAT had an exceptionally low RMSE of 0.6226 along with MAPE of 2.0478% across all the test points. The ANN predicted values correlation coefficient (R) with the original data was 0.987421.

**Fig. 11:**
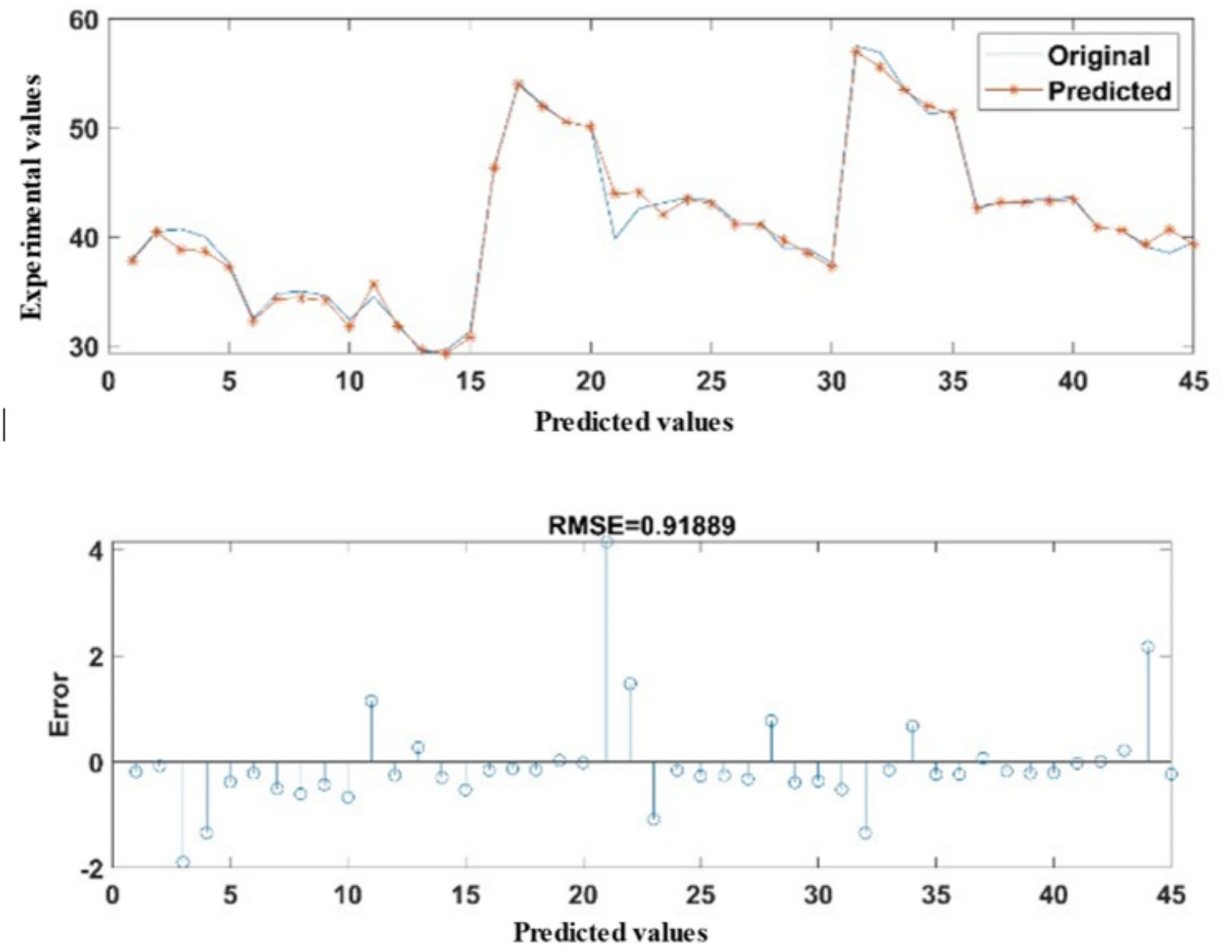
Comparison of ANN predicted New Air Humidity with experimentally measured data.

Similarly, the data for the New Humidity (NAH) were also accurately predicted, as shown by the agreement with the experimental values in all cases of the experimental observations. As illustrates in Fig. 11, the developed ANN model produced an exceptionally low RMSE of 0.9189 in error metrics. The MAPE was observed to be 1.3865%. In the category of correlation metrics, the developed ANN model showed excellent overall agreement indices with the experimental findings with a correlation coefficient (R) of 0.991413. The developed model exhibits exceptional generalisation capabilities, indicating its potential as a useful tool for system control. Validation metrics such as root mean square error (RMSE) and mean absolute percentage error (MAPE) were used to assess model accuracy. For example, the ANN model achieved an RMSE of 0.6226 and a MAPE of 2.05% for temperature predictions, demonstrating excellent agreement with experimental results.

## Conclusions

This investigational study demonstrated the effectiveness of the liquid desiccant system using CaCl_2_ as a TCF for removing moisture from the air, by assessing the influence of operating temperatures on air properties (temperature, RH, and moisture content) and system effectiveness. The following conclusions were drawn from the experimental study.

- Heat energy from a CHP system was used to power cooling through a TCF air conditioning unit and was found to be enough to drive the dehumidification system.
- In the tests performed typically the system reduced air temperature up to 5 °C and over 6–10% RH.
- The dehumidification effectiveness of the system ranges approximately between 65 and 77% for a 5 m/s air mass flow rate and 53–72% for a 12 m/s air mass flow rate. To sum up, it is not recommended to use an air mass flow rate above 12 m/s as it decreases the effectiveness of the system.
- According to the parametric analyses, an air mass flow of 5 m/s, a hot tank temperature of 45 °C, and an inlet solution concentration of 40% are recommended as the best working conditions considering the ecological and economic effects.

An ANN metamodel-based control strategy was proposed and was a promising approach for implementing hybrid thermo-chemical networks, and the performance of the proposed model was validated using various measurement methods. The proposed ANN model achieved a high level of accuracy, indicating its potential for use in real-time applications. The results of this study can be useful for designing and optimising liquid desiccant systems used in air conditioning applications. In summary, the integration of ANN-based control strategies will not only enhance the overall effectiveness of the liquid desiccant system but also provides a robust means of optimising and maintaining system performance in real-world scenarios.

## Data Availability

Our research data are published in the Durham University Research Data Repository. DOI: http://doi.org/10.15128/r1st74cq558.
